# Affective problems and decline in cognitive state in older adults: a systematic review and meta-analysis

**DOI:** 10.1017/S0033291718001137

**Published:** 2018-05-24

**Authors:** A. John, U. Patel, J. Rusted, M. Richards, D. Gaysina

**Affiliations:** 1 EDGE Lab, School of Psychology, University of Sussex, Brighton, UK; 2 School of Psychology, University of Sussex, Brighton, UK; 3 MRC Unit for Lifelong Health and Ageing at UCL, London, UK

**Keywords:** Affective problems, ageing, anxiety, cognitive decline, depression, meta-analysis, systematic review

## Abstract

Evidence suggests that affective problems, such as depression and anxiety, increase risk for late-life dementia. However, the extent to which affective problems influence cognitive decline, even many years prior to clinical diagnosis of dementia, is not clear. The present study systematically reviews and synthesises the evidence for the association between affective problems and decline in cognitive state (i.e., decline in non-specific cognitive function) in older adults. An electronic search of PubMed, PsycInfo, Cochrane, and ScienceDirect was conducted to identify studies of the association between depression and anxiety separately and decline in cognitive state. Key inclusion criteria were prospective, longitudinal designs with a minimum follow-up period of 1 year. Data extraction and methodological quality assessment using the STROBE checklist were conducted independently by two raters. A total of 34 studies (*n* = 71 244) met eligibility criteria, with 32 studies measuring depression (*n* = 68 793), and five measuring anxiety (*n* = 4698). A multi-level meta-analysis revealed that depression assessed as a binary predictor (OR 1.36, 95% CI 1.05–1.76, *p* = 0.02) or a continuous predictor (*B* = −0.008, 95% CI −0.015 to −0.002, *p* = 0.012; OR 0.992, 95% CI 0.985–0.998) was significantly associated with decline in cognitive state. The number of anxiety studies was insufficient for meta-analysis, and they are described in a narrative review. Results of the present study improve current understanding of the temporal nature of the association between affective problems and decline in cognitive state. They also suggest that cognitive function may need to be monitored closely in individuals with affective disorders, as these individuals may be at particular risk of greater cognitive decline.

## Introduction

Decline in cognitive state is a central feature of ageing, and severe deterioration in cognitive function has frequently been associated with poorer quality of life and worse performance on physical tasks (Tabbarah *et al.*, 2002). Accelerated decline in cognitive state also has an influential and adverse impact upon the psychological, social, emotional and financial status of the individual, which can subsequently contribute to heightened levels of burden and distress (Wilson *et al.*, 2007). Cognitive symptoms are common in affective disorders, particularly impairments in memory, executive control, feedback sensitivity and affective processing (Clark *et al.*, 2009). These cognitive symptoms are associated with pathophysiology across a distributed neural circuit, which is made up of various regions across the prefrontal cortex, as well as subcortical regions and also temporal lobe structures (Clark *et al.*, 2009). Both affective disorders, such as depression and anxiety, and poor cognitive function are common in older adulthood (Rovner *et al.*, 1989; Alexopoulos and Abrams, 1991). It is estimated that after age 70, the combination of low mood and poor cognition doubles with every 5 years. By age 85, around one in four of individual experience both these comorbid conditions (Arve *et al.*, 1999). Due to the high prevalence of these conditions in older adulthood, this is a research area of clinical relevance and importance.

Previous research has proposed that affective problems, such as depression and anxiety, may be associated with accelerated cognitive ageing (da Silva *et al.*, 2013; Gulpers *et al.*, 2016). However, there are significant gaps in our understanding of this link. For instance, the precise temporal order of the association between affective problems and decline in cognitive state is currently unclear. It is possible that affective problems may act as an early risk factor for decline in cognitive state, or alternatively that affective problems may be a prodromal symptom of oncoming cognitive impairment. Additionally, previous studies, including several meta-analyses, have been largely diagnosis driven, with a primary focus on dementia as an outcome (Jorm, 2001; Ownby *et al.*, 2006; Byers and Yaffe, 2011; da Silva *et al.*, 2013; Bennett and Thomas, 2014; Cherbuin *et al.*, 2015). Less is known about the impact of affective problems on decline in cognitive state across the entire population spectrum. The focus on the transition to dementia as an outcome may be problematic, as it is now believed that there is a long pre-clinical period of several decades before cognitive impairment becomes evident (Morris, 2005). It is possible that participants who transition to dementia at follow-up assessment may have already developed substantial cerebral pathology by the time of baseline assessment, even if they were not yet presented with any cognitive symptoms. In this case, associations between affective disorders and development of dementia may be the result of reverse causality. The present study focuses on the association between affective disorders and decline in cognitive state in healthy older adults in order to minimise effects of possible reverse causality.

Cognitive state refers to a composite measure of overall cognitive function. It has been studied extensively in previous research (Nordin *et al.*, 2006; Kavé *et al.*, 2008; Sohrabi *et al.*, 2008; Esslinger *et al.*, 2011), using assessments of overall cognitive status, such as the Mini-Mental State Examination (MMSE) or composite assessments of multiple cognitive domains (e.g. memory, information processing speed, executive function). Therefore, decline in cognitive state is defined in the present review as a decline in overall cognitive function, rather than decline in specific cognitive domains. There is evidence from longitudinal research that low scores on cognitive state tests may predict onset of functional impairment (Moritz *et al.*, 1995; Gill *et al.*, 1996), and functional dependence over time (Agüero-Torres *et al.*, 2002; Gill *et al.*, 2002; Wang *et al.*, 2002). For this reason, it is important to investigate how affective problems influence decline in cognitive state over time.

There are large individual differences in the extent of cognitive decline experienced by healthy older adults; however, the decline in cognitive state occurs at a steady and gradual rate over time. On average there is a decline of around 1–2 standard deviations in fluid cognition from age 20 to 70, after which average decline increases to around 0.5 s.d. every 10 years (Anstey and Low, 2004). This stable decline is often maintained over time until symptoms of dementia begin to manifest, at which point a sharper decline in cognitive state may be observed (Rubin *et al.*, 1998). As such, studies in which substantial cognitive decline is apparent within a short time frame of under 1 year may be more indicative of pathological ageing (e.g. oncoming dementia), rather than healthy ageing. Since the present study aims to examine the longitudinal association between affective disorders and decline in cognitive state in cognitively healthy individuals, it includes only longitudinal studies with sufficient time between baseline and follow-up assessments (i.e., minimum 1 year) for a substantial decline to occur within these populations. There are several studies that have investigated the association between affective problems and decline in cognitive state (Bassuk *et al.*, 1998; Geerlings *et al.*, 2000; Paterniti *et al.*, 2002; Ganguli *et al.*, 2006; Reyes-Ortiz *et al.*, 2008; Köhler *et al.*, 2010; Bunce *et al.*, 2012; Gale *et al.*, 2012; Johnson *et al.*, 2013; Neubauer *et al.*, 2013; Royall and Palmer, 2013; Rajan *et al.*, 2014; Chang and Tsai, 2015, Chen and Chang, 2016; Brailean *et al.*, 2017). However, it is difficult to draw a straightforward conclusion from this work due to conflicting findings. Specifically, some studies report a significant association between affective problems and decline in cognitive state (Geerlings *et al.*, 2000; Paterniti *et al.*, 2002; Reyes-Ortiz *et al.*, 2008; Köhler *et al.*, 2010; Johnson *et al.*, 2013; Royall and Palmer, 2013; Rajan *et al.*, 2014; Chang and Tsai, 2015, Chen and Chang, 2016), while conversely others report that affective problems do not predict decline (Bassuk *et al.*, 1998; Ganguli *et al.*, 2006; Bunce *et al.*, 2012; Gale *et al.*, 2012; Neubauer *et al.*, 2013; Brailean *et al.*, 2017). These contradictory results are likely attributable to inconsistencies in methodologies and study design, such as length of follow-up, sampling, definitions used, differences in assessment tools and also the primary aim of each study (Bennett and Thomas, 2014). To date, however, there have been no systematic reviews or meta-analyses addressing associations between affective problems and subsequent decline in cognitive state, prior to the onset of dementia. Due to inconsistencies in findings, as well as the lack of attempts to synthesise these data, it is still unclear whether affective problems across the life course are associated with a decline in cognitive state, prior to the onset of dementia and cognitive impairment. The primary aim of the present study therefore was to systematically review and synthesise current evidence regarding the longitudinal association between affective problems (depression and anxiety separately) and subsequent decline in cognitive state, with consideration of several potential moderators, including mean age of sample at baseline, length of follow-up, quality of study and publication year.

## Method

### Search strategy

This review was conducted in accordance with the Preferred Reporting Items for Systematic reviews and Meta-Analyses (PRISMA) guidelines (Moher *et al.*, 2009). A systematic literature search was conducted using PubMed, PsycInfo, Cochrane, and ScienceDirect databases for studies investigating the association between affective problems and decline in cognitive state. All studies published up to November 2016 were included in the search. There was no restriction on the start date. Our search terms comprised three search blocks (Table 1). The first search block included keywords relating to affective problems. The second search block contained keywords describing the decline in cognitive state. To reduce the number of irrelevant hits, a third search block was added, which contained keywords related to methodology, to ensure all studies with cross-sectional designs were excluded from search results. In addition, reference lists of relevant papers were scanned for articles of interest.

**Table 1.** Key terms used for systematic search

Search block 1 (Affective problems)	Search block 2 (Cognitive decline)	Search block 3 (Study design)
Depress* OR MDD OR Dysthymi* OR Anxi* OR GAD OR worr* OR Phobia OR Panic OR Agoraphobia OR ‘Obsessive compulsive’ OR OCD OR PTSD OR ‘Post traumatic stress’ OR ‘Post-traumatic stress’ OR mood OR affective OR psychiatric OR neuropsychiatric	‘Cognitive function’ OR ‘Cognitive impairment’ OR ‘Cognitive decline’ OR ‘Cognitive deficit’ OR ‘Cognitive loss’ OR ‘Cognition loss’ OR ‘Cognitive ability’ OR ‘Cognitive abilities’ OR ‘Cognitive status’ OR ‘Cognitive change’ OR ‘Cognition change’ OR ‘Cognitive performance’ OR ‘Cognitive dysfunction’ OR ‘Cognitive complaints’ OR ‘Cognitive capability’ OR ‘Cognitive ageing’ OR ‘Cognitive aging’ OR Memory OR Attention OR ‘Reaction time’ OR ‘Speed of processing’ OR ‘Processing speed’ OR Intelligence OR ‘General mental ability’ OR GMA OR ‘Executive function’ OR ‘Neuropsychological testing’ OR ‘Mini mental state exam’ OR MMSE OR ‘Mental status’	Longitudinal OR prospective OR follow-up OR cohort OR ‘life course’ OR lifespan OR ‘life span’ OR lifelong OR ‘lifelong’

### Inclusion/exclusion criteria

Stringent inclusion/exclusion criteria were applied to articles identified through the initial search.

#### Design criteria

Original studies written in English up to November 2016 were included. Only studies using longitudinal, prospective designs with human participants were included in order to test for the association between affective disorders and decline in cognitive state over time. Cross-sectional, case-control, experimental, including intervention and treatment, studies were excluded. Studies with a follow-up period of 1 year or greater were included, as it is possible that substantial decline in cognitive state may not be observed over very short follow-up periods in a general population. Included studies used samples drawn from a general population, whereas studies using specific clinical populations only, for example, a sample of stroke patients, were excluded. This criterion was used because inclusion of clinical samples may increase the heterogeneity of data synthesis and reduce the comparability of studies. Studies with a sample size of 100 or less were also considered ineligible, due to insufficient statistical power.

#### Outcome-related criteria

Samples with cognitive impairment or dementia present at baseline were excluded. In addition to this, studies with any measure of change in cognitive state from baseline to follow-up were selected for inclusion. Other outcomes, such as the transition to dementia or cognitive performance at follow-up without consideration of change from a baseline measure were omitted. This was because the present study aimed to look at the association between affective disorders and cognitive decline within healthy ageing populations, rather than samples with dementia. Additionally, studies assessing specific cognitive domains, such as attention or visuospatial ability exclusively, rather than cognitive state, were also excluded to reduce heterogeneity.

#### Predictor-related criteria

Both diagnostic and dimensional measures of depression and anxiety at baseline assessment were judged as eligible. Studies with retrospective assessments of affective problems were excluded, as such assessments may be less reliable. Both binary indicators of affective problems, defined as either a diagnosis or as a score above a threshold level, or continuous symptoms scores, as assessed by a validated scale of affective problems were included in this review.

### Screening procedure

All articles identified through our search strategy were screened for eligibility using a three-step process. All references were first reviewed by title. Next, the remaining references were screened by abstract. Finally, all remaining articles were read in full and final eligibility determinations were made on this basis. All articles were reviewed for inclusion by one rater, and 10% of all articles were additionally screened by an independent rater, in order to assess the consistency of screening. Any disagreements were resolved during consensus meetings.

### Data extraction

Data from the relevant articles were extracted using a detailed coding form. Information extracted included: Study information (Authors, publication year, DOI); Sample information (Country, mean age at baseline, gender composition, ethnicity, year of data collection, number of follow-ups, time between lags, total length of follow-up, sample size at baseline, sample size at final follow-up); Instrument information (Type of affective problem, measure used to assess affective problem, measure used to assess decline in cognitive state); Statistical information (Statistical test used, effect sizes, covariates adjusted for in statistical model). Where results for more than one follow-up were reported, the longest follow-up was selected for our analysis, as longer follow-up times allow a greater period for the decline in cognitive state to occur. Similarly, where multiple models were reported with various adjustments made, the most conservative model (with the greatest amount of adjustments) was selected. In cases where insufficient statistical information was available, original authors were contacted directly via email. All studies were evaluated for methodological quality using STROBE (Strengthening the Reporting of Observational Studies in Epidemiology) guidelines (Elm *et al.*, 2007).

### Statistical analysis and data synthesis

All analyses were conducted in R Studio (Studio, 2012), using the *metafor* package. Separate meta-analyses were run for studies in which affective problems were assessed as a binary predictor (using a defined threshold), and studies where affective problems were assessed as a continuous predictor (using a symptom score). In addition, separate analyses were conducted for studies that used depression or anxiety as predictors of decline in cognitive state.

Odds ratios (ORs) were used as a common effect size across studies with a binary measure of affective problems. When ORs were not reported in original studies, these were estimated from available data using standard computational techniques (Lipsey and Wilson, 2000; Borenstein *et al.*, 2009; Field and Gillett, 2010). Log ORs were then computed for subsequent analysis. Standardised regression coefficients were used as a common effect size across studies with a continuous measure of affective problems. If unstandardised effect sizes were reported, or measures were not standardised to a *z* score before analysis, coefficients were converted to standardised coefficients using standard computational methods (Kim and Ferree, 1981; Duncan, 2014). In cases where insufficient information was reported in the study to calculate the standardised coefficients, authors were contacted directly via email. We also converted the estimated regression coefficients into ORs to facilitate the comparison with the analyses using a binary predictor of depression.

Multi-level meta-analyses were conducted to account for multiple effect sizes within studies (Van Den Noortgate and Onghena, 2003). Heterogeneity across studies was assessed using the *Q* statistic, with *p* < 0.1 suggesting significant heterogeneity between studies, and the *I*^2^ statistic, in which 25, 50 and 75% represent low, medium and high heterogeneity (Higgins *et al.*, 2003).

Additional meta-regression analyses were also conducted to assess the effects of potential moderators, including the length of follow-up, age of sample at baseline, publication year, method of affective problem assessment (diagnosis or self-report) and quality of studies. All moderators were entered initially as continuous variables, except for the assessment method of affective problems which was coded as a binary variable. For significant moderators, binary variables were created using the average values and sub-group analyses were run using these variables.

Publication bias was assessed using Begg's funnel plot and Begg's rank correlation test (Richard and Pillemer, 1984; Song *et al.*, 2000; Egger *et al.*, 2008).

## Results

### Literature search

**Fig. 1.**
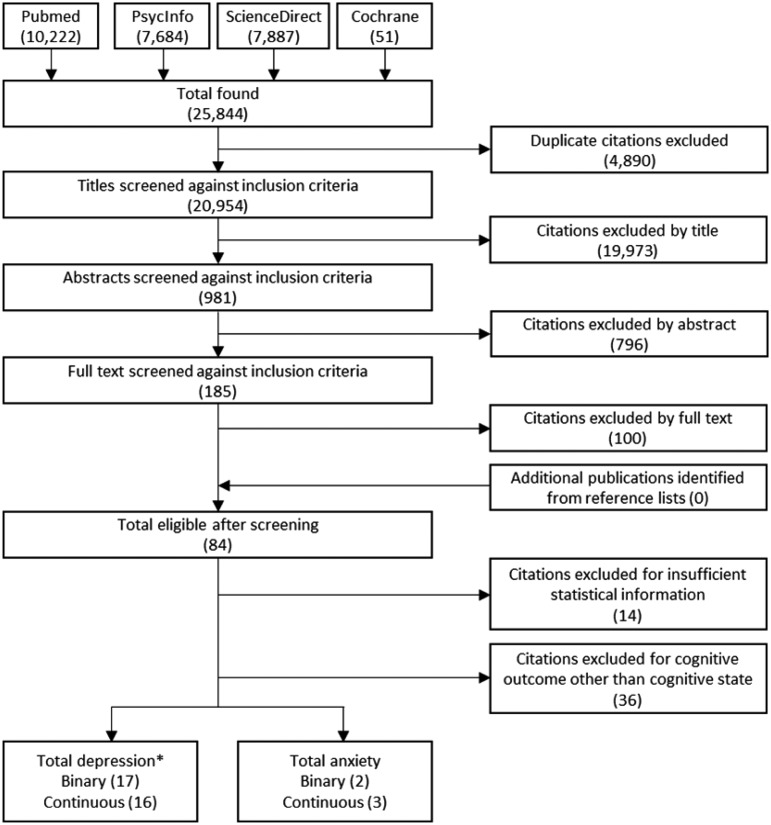
Flowchart of selection.

*Note*: *One study assessed both binary and continuous assessments of depression, meaning that although there are 17 studies using binary measures of depression and 16 studies using continuous measures of depression, in total there are only 32 studies reporting on depression and cognitive decline.

Our search identified 25 844 references. After exclusion of duplicates, 20 954 unique citations remained. At stage 1, citations were screened by title and 981 were determined to be eligible (inter-rater reliability = 96%). In stage 2, remaining citations were screened by abstract and 185 were judged as relevant (inter-rater reliability = 91%). Finally, all 185 citations were selected for full-text screening, after which 84 references remained (inter-rater reliability = 94%). At this stage, a further 36 studies were excluded, as they were addressing the decline in specific cognitive outcomes, rather than cognitive state. Of the 48 studies remaining, initially there were 18 with insufficient information for calculation of effect sizes. Authors were contacted directly by email about this and four (22%) responded to provide the relevant information. This left a total of 34 studies with sufficient information to calculate effect sizes, with 32 studies investigating the link between depression and a decline in cognitive state (*n* = 68 793), and five studies investigating anxiety and decline in cognitive state (*n* = 4698; Fig. 1 and Table 2).

**Table 2.** Studies included in the systematic literature review and meta-analyses

Study ID	Author	Year	Country	% Female	Mean age at baseline	Mean length of follow-up	Measure of cognition	Type of affective problem	Measure of affective problem
1	Bassuk	1998	USA	63	73.72	12	Short Portable Mental Status Questionnaire (SPMSQ)	Depression	Center for Epidemiologic Studies Depression Scale (CES-D)
2	Brodaty	2012	Australia	59	78.41	2	Mean of other cognitive domain scores	Depression	Neuropsychiatric Inventory
2	Brodaty	2012	Australia	59	78.41	2	Mean of other cognitive domain scores	Anxiety	Neuropsychiatric Inventory
3	Chang	2015	Taiwan	49	63.34	4	Short Portable Mental Status Questionnaire (SPMSQ)	Depression	Center for Epidemiologic Studies Depression Scale (CES-D)
4	Downer	2016	USA	58	73.18	14	Mini Mental state Examination (MMSE)	Depression	Center for Epidemiologic Studies Depression Scale (CES-D)
5	Dufouil	1996	France	60	74.78	3	Mini Mental state Examination (MMSE)	Depression	Center for Epidemiologic Studies Depression Scale (CES-D)
6	Ganguli	2006	USA	61	74.60	12	Mini Mental state Examination (MMSE)	Depression	Modified Center for Epidemiologic Studies Depression Scale (CES-D)
7	Geerlings	2000	The Netherlands	51	69.39	3	Mini Mental state Examination (MMSE)	Depression	Center for Epidemiologic Studies Depression Scale (CES-D) (>16 vs. <16)
8	Han	2008	Canada	66	79.11	1	Mini Mental state Examination (MMSE)	Depression	Hamilton Depression Rating Scale (HDRS)
9	Kohler	2010	The Netherlands	48	69.40	6	Mini Mental state Examination (MMSE)	Depression	Revised 90-item version of the Symptom Checklist (SCL-90)
10	Niti	2009	Singapore	64	65.40	1.5	Mini Mental state Examination (MMSE)	Depression	Chinese version of the 15 item Geriatric Depression Scale (GDS)
11	Paterniti	2002	France	43	64.96	4	Mini Mental state Examination (MMSE)	Depression	Center for Epidemiologic Studies Depression Scale (CES-D)
12	Raji	2007	USA	59	72.70	7	Mini Mental state Examination (MMSE)	Depression	Center for Epidemiologic Studies Depression Scale (CES-D)
13	Reyes-Ortiz	2008	USA	57	72.70	11	Mini Mental state Examination (MMSE)	Depression	Center for Epidemiologic Studies Depression Scale (CES-D)
14	Rosenblatt	2003	USA	63	40.30	11.5	Mini Mental state Examination (MMSE)	Depression	Diagnostic Interview Schedule (DIS)
15	Sawyer	2012	USA	69	70.18	4	Mini Mental state Examination (MMSE)	Depression	Duke Depression Evaluation Schedule (DDES)
16	Sinoff	2003	Israel	63	77.64	3.1	Mini Mental state Examination (MMSE)	Anxiety	Sinoff's Short Anxiety Screening Test (SAST)
17	Wilson	2016	USA	74	76.30	8	Battery of 19 cognitive performance tests	Depression	Subset of questions from the Diagnostic Interview Schedule
18	Yaffe	1999	USA	100	72.94	4	Mini Mental state Examination (MMSE)	Depression	Geriatric Depression Scale (GDS)
19	Bierman	2008	The Netherlands	53	69.49	9	Mini Mental state Examination (MMSE)	Anxiety	Hospital Anxiety and Depression Scale-Anxiety (HADS-A)
20	Bunce	2012	Australia	49	76.55	12	Mini Mental state Examination (MMSE)	Depression	Goldberg Depression Scale
20	Bunce	2012	Australia	49	76.55	12	Mini Mental state Examination (MMSE)	Anxiety	Goldberg Anxiety Scale
21	Chen	2016	Taiwan	45	70.95	14	Short Portable Mental Status Questionnaire (SPMSQ)	Depression	Center for Epidemiologic Studies Depression Scale (CES-D)
22	Chiao	2016	Taiwan	57	71.04	14	Short Portable Mental Status Questionnaire (SPMSQ)	Depression	Center for Epidemiologic Studies Depression Scale (CES-D)
23	Dotson	2008	USA	40	75.38	4.4	Mini Mental State Exam (MMSE), and Blessed Information Memory and Concentration Scale (BIMCS)	Depression	Center for Epidemiologic Studies Depression Scale (CES-D)
24	Gale	2012	England	55	64.07	6	Principal components analyses of 5 cognitive outcomes	Depression	Center for Epidemiologic Studies Depression Scale (CES-D)
25	Geerlings	2000	The Netherlands	51	69.39	3	Mini Mental state Examination (MMSE)	Depression	Center for Epidemiologic Studies Depression Scale (CES-D) score (per point increase)
26	Han	2006	Canada	66	79.11	1	Mini Mental state Examination (MMSE)	Depression	HDRS
27	Johnson	2013	USA	69	73.50	2	Mini Mental state Examination (MMSE) & Clinical Dementia Rating (CDR-SB)	Depression	Geriatric Depression Scale (GDS30)
28	Neubauer	2013	Germany	60	75.70	1	Mini Mental state Examination (MMSE) and Syndrome Short Test (SKT)	Depression	Geriatric Depression Scale (GDS30)
29	Panza	2009	Italy	36	71.90	3.5	Mini Mental state Examination (MMSE)	Depression	Italian version of the Geriatric Depression Scale (GDS)
30	Rajan	2014	USA	63	72.41	9	Cognitive battery of four tests	Depression	Center for Epidemiologic Studies Depression Scale (CES-D)
31	Royall	2013	Japan, Hawaii, and the mainland-US		77.80	10	Cognitive Abilities Screening Instrument (CASI)	Depression	Center for Epidemiologic Studies Depression Scale (CES-D)
32	Turner	2015	USA	70	73.90	5	Measure derived from 19 cognitive tests	Depression	Center for Epidemiologic Studies Depression Scale (CES-D) & Geriatric Depression Scale (GDS)
33	Van den Kommer	2013	The Netherlands	53	69.25	13	Mini Mental state Examination (MMSE)	Depression	Center for Epidemiologic Studies Depression Scale (CES-D)
34	Vinkers	2004	The Netherlands	63	85.00	4	Mini Mental state Examination (MMSE)	Depression	Geriatric Depression Scale (GDS)
35	Wilson	2011	USA	76	80.70	3.4	Derived from 19 cognitive tests	Depression	48-item Neuroticism scale from the NEO Personality Inventory-Revised. Depression sub-scale
35	Wilson	2011	USA	76	80.70	3.4	Derived from 19 cognitive tests	Anxiety	48-item Neuroticism scale from the NEO Personality Inventory-Revised. Anxiety sub-scale

#### Depression studies

Of the depression studies, 17 used a binary measure of depression (*k* = 34) and 16 measured depression as a continuous variable (*k* = 36). Depression studies had a mean follow-up length of approximately 6.61 years (s.d. = 4.41). The mean age of participants was 72.15 at baseline (s.d. = 7.56) and the gender composition of the sample was approximately 59.48% female. The majority of studies took place in the USA (*n* = 14), followed by the Netherlands (*n* = 4) and Taiwan (*n* = 3). Studies also took place in Australia, Canada, France (*n* = 2 for each), Germany, England, Italy, Singapore and Japan, Hawaii and the mainland-USA (*n* = 1 for each). Overall, the majority of studies used the Center for Epidemiologic Studies Depression Scale (CES-D) to assess depression present at baseline (*n* = 16), followed by the Geriatric Depression Scale (GDS) (*n* = 7), the Diagnostic Interview Schedule (DIS) (*n* = 3), the Neuroticism scale from the NEO Personality Inventory, the Duke Depression Evaluation Schedule (DDES), the Goldberg Depression Scale, Neuropsychiatric Inventory, Hamilton Rating Scale for Depression (HDRS), the Symptom Checklist (*n* = 1 for each). Only one study reported separate effect sizes for more than one follow-up period (Bassuk *et al.*, 1998). This study reported effect sizes at 3 years after baseline (*n* = 2030), 6 years after baseline (*n* = 1447) and 12 years after baseline (*n* = 756). The effect size with the longest follow-up (12 years) was selected for inclusion in the meta-analysis.

#### Anxiety studies

Of the five anxiety studies, two used a binary indicator of anxiety (*k* = 2) and three used a continuous measure of anxiety (*k* = 3). Anxiety studies had a mean follow-up time of 5.9 years (s.d. = 4.36). On average, participants were 76.56 years old at baseline (s.d. = 4.23) and were predominantly female (60.14% female). The majority of studies took place in Australia (*n* = 2), followed by the USA, the Netherlands and Israel (*n* = 1 for each). Anxiety was assessed using Sinoff's Short Anxiety Screening Test (SAST), Hospital Anxiety and Depression Scale-Anxiety (HADS-A), Neuropsychiatric Inventory, Goldberg Anxiety Scale and the Neuroticism scale from the NEO Personality Inventory (*n* = 1 for each). All anxiety studies had a score of 60% or greater on the STROBE checklist (maximum score = 81%, median score = 78%).

### Depression and decline in cognitive state

#### Meta-analysis of studies with depression as a binary predictor

**Fig. 2.**
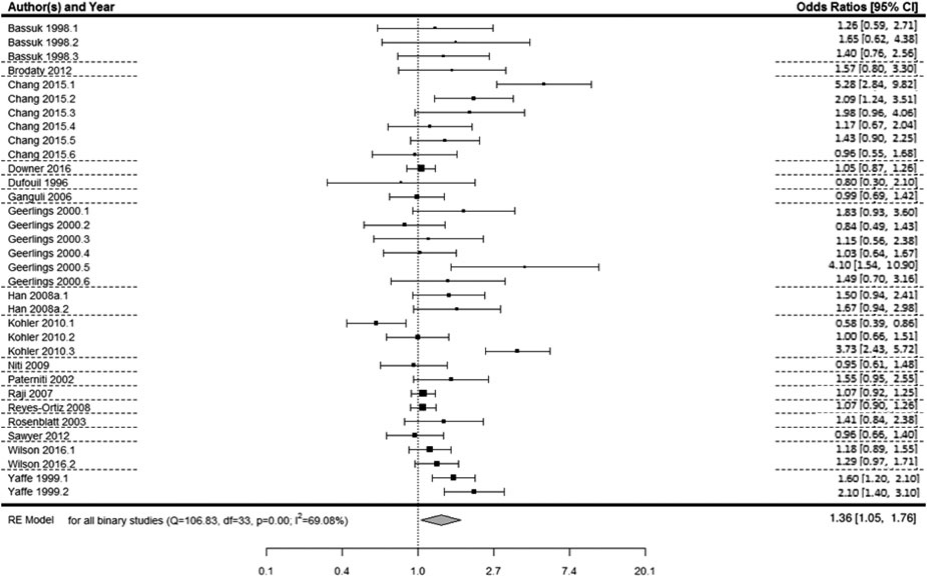
Forest plot of the association between binary depression and decline in cognitive state*. Notes for multiple effect sizes within studies: Bassuk* (1: High SPMSQ at baseline; 2: Medium SPMSQ at baseline; 3: High or medium SPMSQ at baseline), Chang (1: Males with persistent depressive symptoms; 2: Males with increasing depressive symptoms; 3: Males with decreasing depressive symptoms; 4. Females with persistent depressive symptoms; 5: Females with increasing depressive symptoms; 6: Females with decreasing depressive symptoms), Geerlings* (1: CES-D threshold in high education sample; 2: CES-D threshold in low education sample; 3: Felt depressed some of the time *v.* never in high education sample; 4: Felt depressed some of the time *v.* never in low education sample; 5: Felt depressed most of the time *v.* never in high education sample; 6: Felt depressed most of the time *v.* never in low education sample), Han (1: Major depression *v.* no depression; 2: Minor depression *v.* no depression), Kohler (1: Low depression *v.* no depression; 2: Middle depression *v.* no depression; 3: High depression *v.* no depression), Wilson* (1: Major depression *v.* no depression; 2: Elevated depression symptoms *v.* no depression), Yaffe (1: 3–5 depressive symptoms *v.* 0–2 depressive symptoms; 2: >6 depressive symptoms *v.* 0–2 depressive symptoms).

There were 34 relevant effect sizes across 17 studies with a binary measure of depression (Dufouil *et al.*, 1996; Bassuk *et al.*, 1998; Yaffe *et al.*, 1999; Geerlings *et al.*, 2000; Paterniti *et al.*, 2002; Rosenblatt *et al.*, 2003; Ganguli *et al.*, 2006; Raji *et al.*, 2007; Han *et al.*, 2008; Reyes-Ortiz *et al.*, 2008; Niti *et al.*, 2009; Köhler *et al.*, 2010; Brodaty *et al.*, 2012; Sawyer *et al.*, 2012; Chang and Tsai, 2015; Downer *et al.*, 2016; Wilson *et al.*, 2016). A multi-level meta-analysis of 34 effect sizes revealed that depression was associated with an increased risk of subsequent decline in cognitive state (OR 1.36, 95% CI 1.05–1.76, *p* = .02; Fig. 2).

* Represents effect sizes within studies where there may be some overlap in the sample.

#### Assessment of heterogeneity, meta-regression and sub-group analyses

Significant heterogeneity was observed across the studies with depression as a binary predictor (*Q* = 106.83, df = 33, *p* < 0.0001, *I*^2^ = 69.08%). An omnibus meta-regression analysis including publication year, mean age at baseline, length of follow-up, method of depression assessment (diagnosis or self-report) and quality of study revealed that these variables together were able to explain a significant amount of heterogeneity in the model (QM = 13.32, df = 5, *p* = 0.02). However, even after accounting for these factors, significant heterogeneity remained in the model (QE = 93.51, df = 28, *p* < 0.0001). To further explore the effect of publication year, age at baseline, length of follow-up, depression assessment and quality on heterogeneity, individual meta-regressions were conducted for each of these potential modifiers. These analyses revealed that mean age at baseline (*p* = 0.13), publication year (*p* = 0.19), quality of the study (*p* = 0.09) and depression assessment (*p* = 0.91) did not significantly explain the between-study variability.

Meta-regression analyses including the length of follow-up showed significant between-study variability, whereby studies with shorter follow-up periods had significantly greater effect sizes than studies with longer follow-up periods (*B* = −0.03, s.e. = 0.009, *p* = 0.002). Additionally, meta-regression analyses including the method of cognitive assessment (MMSE *v.* neuropsychiatric batteries) showed significant between-study variability (*B* = −0.2, s.e. = 0.08, *p* = 0.01). To further explore precisely how these significant factors were involved in this association sub-group meta-analyses were conducted.

To explore how length of follow-up affected the association, effect sizes were divided by the mean follow-up length in years (M = 6.35 (s.d. = 4.25) years), resulting in two groups of longer (*k* = 7, M = 10.79 (s.d. = 2.45) years) and shorter follow-up periods (*k* = 10, M = 3.25 (s.d. = 1.48) years). Multi-level sub-group meta-analyses revealed that depression was significantly associated with decline in cognitive state in studies with shorter follow-up periods (OR 1.43, 95% CI 1.03–2.00, *p* = 0.03) and was approaching significance in studies with longer follow-up periods (OR 1.15, 95% CI 0.98–1.36, *p* = 0.08). However, the overall effect size was larger for studies with shorter follow-up periods than those with longer follow-up periods. Studies with longer follow-up periods did not differ significantly from studies with shorter follow-up periods on quality (*t*_(14.29)_ = 1.08, *p* = 0.3), publication year (*t*_(15.38)_ = 0.1, *p* = 0.92), mean age at baseline (*t*_(10.67)_ = −0.41, *p* = 0.69), or depression assessment (*t*_(14.07)_ = −0.5, *p* = 0.63).

The meta-regression analysis including the method of cognitive assessment suggested that effect sizes were significantly smaller for studies using the MMSE than studies using neuropsychiatric batteries (*B* = −0.2, s.e. = 0.08, *p* = 0.01). However, there were only four studies using neuropsychiatric battery assessments of cognitive state, so results need to be treated with caution.

#### Meta-analysis of studies with depression as a continuous predictor

**Fig. 3.**
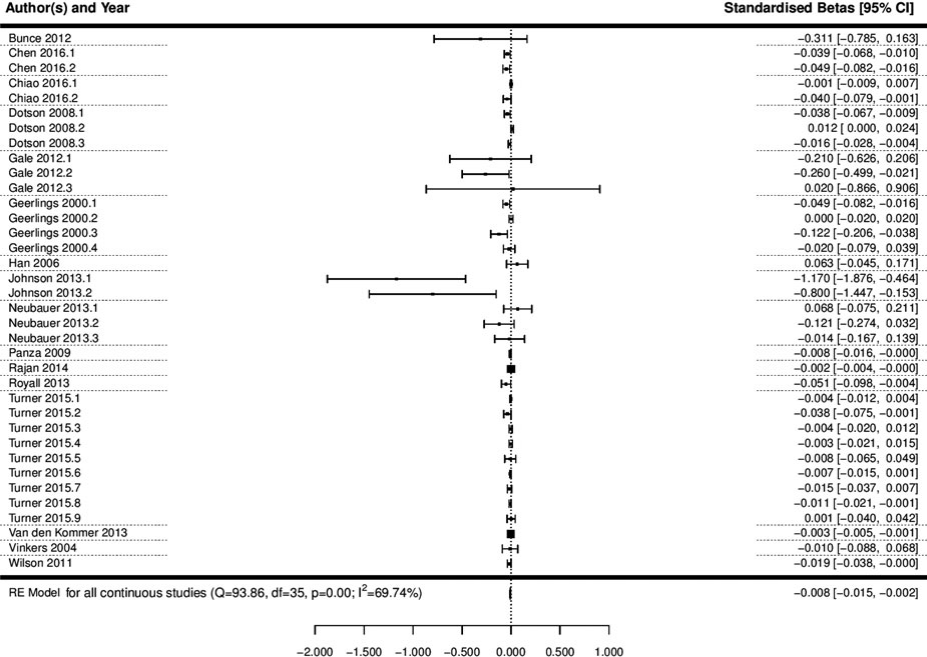
Forest plot of the association between continuous depression and a decline in cognitive state. Notes for multiple effect sizes within studies: Chen 2016 (1: Cognition starting high and declining; 2: Cognition starting low and declining), Chiao 2016* (1: Negative affect; 2: Lack of positive affect), Dotson 2008* (1: Baseline CES-D on MMSE; 2: Average CES-D on BIMCS; 3: Average CES-D on MMSE); Gale 2012 (1: Age 50–60; 2: Age 60–80; 3: Age 80–90), Geerlings 2000* (1: CES-D Score per point increase, education >8 years; 2: CES-D Score per point increase, education < 8 years; 3: Negative affect score per point increase, education >8 years; 4: Negative affect score per point increase, education <8 years), Johnson 2013* (1: MMSE; 2: CDR-SB), Neubauer 2013* (1: Depression at T1 predicting cognition change from T1 to T2; 2: Depression at T2 predicting cognition change from T2 to T3; 3: Depression at T3 predicting cognition change from T3 to T4), Turner 2015* (1: CES-D; 2: CES-D Positive affect; 3: CES-D Negative affect; 4: CES-D Somatic complaints; 5: CES-D Interpersonal problems; 6: GDS; 7: GDS Positive affect; 8: GDS Negative affect; 9: GDS Positive and negative affect).

A multi-level meta-analysis of the 36 effect sizes across 16 studies with a continuous measure of depression was conducted (Geerlings *et al.*, 2000; Vinkers *et al.*, 2004; Han *et al.*, 2006; Dotson *et al.*, 2008; Panza *et al.*, 2009; Wilson *et al.*, 2011; Bunce *et al.*, 2012; Gale *et al.*, 2012; Johnson *et al.*, 2013; Neubauer *et al.*, 2013; Royall and Palmer, 2013; Van den Kommer *et al.*, 2013; Rajan *et al.*, 2014; Turner *et al.*, 2015; Chen and Chang, 2016; Chiao and Weng, 2016). This analysis revealed that depression was significantly associated with a decline in cognitive state (*B* = −0.008, 95% CI −0.015 to −0.002, *p* = 0.012; Fig. 3; OR 0.992, 95% CI 0.985–0.998).

* Represents effect sizes within studies where there may be some overlap in the sample.

#### Assessment of heterogeneity and meta-regression analyses

Significant heterogeneity was observed across studies with depression as a continuous predictor (*Q* = 93.86, df = 35, *p* < 0.0001, *I*^2^ = 69.74%). In order to try and explain some of this heterogeneity, an omnibus meta-regression analysis was conducted, including mean age at baseline, length of follow-up, quality and publication year as potential moderators. This analysis revealed that together these variables were not able to explain a significant amount of the heterogeneity in the model (QM = 8.97, df = 4, *p* = 0.06), but even after accounting for these factors, significant heterogeneity remained within the model (QE = 84.9, df = 31, *p* < 0.0001). In order to explore the influence of age at baseline, follow-up length, quality and publication year in more depth, individual meta-regressions were conducted for each potential modifier. These analyses revealed that mean age at baseline (*p* = 0.27), length of follow-up (*p* = 0.1), publication year (*p* = 0.18), method of cognitive assessment (*p* = 0.47) and quality (*p* = 0.11) could not significantly explain between-study variability individually.

### Publication bias

Publication bias is unlikely for meta-analyses of depression studies measured as a continuous variable, as Begg's rank correlation test was non-significant (*p* = 0.07). Begg's funnel plot also appears relatively symmetrical (online Supplementary Fig. S1). There may have been some publication bias present in the meta-analysis of studies with depression as a binary predictor, as although Begg's funnel plot appears symmetrical (online Supplementary Fig. S1), Begg's rank correlation test was significant (*p* = 0.02). Results should, therefore, be interpreted with caution.

### Anxiety and decline in cognitive state

Due to the limited number of studies with anxiety which met our inclusion criteria, meta-analyses for these studies were not possible. Instead, these studies are described in the form of a narrative review. Of the five relevant anxiety studies, two used a binary indicator of anxiety (Sinoff and Werner, 2003; Brodaty *et al.*, 2012) and three used a continuous measure of anxiety (Bierman *et al.*, 2008; Wilson *et al.*, 2011; Bunce *et al.*, 2012).

Two of these studies reported that anxiety was a significant predictor of decline in cognitive state (Sinoff and Werner, 2003; Wilson *et al.*, 2011). Specifically, Sinoff and Werner, 2003 reported that in a sample of 100 people, anxiety (assessed using Sinoff's Short Anxiety Screening Test – SAST) had a strong direct and indirect effect on predicting future decline in cognitive state over 3.2 years (*B* = 0.23, 95% CI −0.03 to –3.95, *p* < 0.05). Similarly, Wilson *et al.* (2011) found that in 785 older adults, higher levels of anxiety symptoms (assessed using the anxiety sub-scale from the 48-item Neuroticism scale) were significantly associated with more rapid decline in cognitive state over a 3.4-year period (*B* = −0.003, s.e. = 0.001, *p* = 0.01).

Conversely, three of the eligible studies found no association between anxiety symptoms and a decline in cognitive state (Bierman *et al.*, 2008; Brodaty *et al.*, 2012; Bunce *et al.*, 2012). Bierman *et al.* (2008) found no evidence that anxiety (assessed using the anxiety sub-scale from the Hospital Anxiety and Depression Scale – HADS-A) predicted a linear decline in cognitive state in a sample of 2351 people over a period of 9 years. Instead, a significant negative quadratic trend for cognition was reported. The authors state that this is suggestive of a curvilinear association between anxiety levels and cognitive performance. Specifically, milder anxiety symptoms may be associated with an improvement on the MMSE until it reaches an optimal level, beyond which the beneficial influence reduces, so more severe anxiety is related to poorer cognitive function. The authors posit that the Yerkes and Dodson law regarding the association between arousal and cognitive performance (Yerkes and Dodson, 1908; Mendl, 1999) may also apply to anxiety symptoms. Brodaty *et al.* (2012) found that in a sample of 480 non-impaired people, the odds of decline in global cognitive state over a period of 2 years were not significantly higher for participants with anxiety (assessed using Neuropsychiatric Inventory) at baseline than those without (OR 1.63, 95% CI 0.5–5.8, *p* = 0.45). They did, however, find a significant effect of anxiety at baseline on the decline in executive function (OR 3.54, 95% CI 1.3–9.9, *p* = 0.016). Finally, Bunce *et al.* (2012) found no evidence that anxiety (assessed using the Goldberg Anxiety Scale) affected change in cognitive state over a period of 12 years in a sample of 836 community-dwelling individuals over the age of 70 (*B* = −0.14, s.e. = 0.19, *p* = 0.46).

## Discussion

The aim of the current study was to systematically investigate associations between affective problems (depression and anxiety) present at baseline and subsequent decline in cognitive state. Our findings revealed that individuals with depression (measured as a binary or continuous predictor) were at an increased risk of a greater decline in cognitive state. These findings are consistent with previous reviews which have indicated an association between affective problems and development of dementia (Jorm, 2001; Ownby *et al.*, 2006; Byers and Yaffe, 2011; da Silva *et al.*, 2013; Bennett and Thomas, 2014; Cherbuin *et al.*, 2015; Gulpers *et al.*, 2016). Our results extend these findings by linking affective problems to a greater decline in cognitive state in samples without dementia at baseline.

### Strengths and limitations

Several limitations of the current study must be acknowledged. They are subject to the limitations of the included studies. Our analyses suggest that there are several key methodological differences between studies which significantly affect the results produced. For example, our results suggest that effects may differ based on length of follow-up. As shown, this is unrelated to differences in publication year, age at baseline, or method of depression assessment (self-report or diagnosis). It is possible that this is more likely attributable to additional unobserved heterogeneity.

This review only included studies of decline in cognitive state as an outcome. For this reason, it is not clear whether affective problems may differentially influence decline in different cognitive domains. Additionally, the majority of included studies assessed cognitive state using the MMSE. This measure has been criticised for lacking sensitivity to subtle changes in cognition and for ceiling and floor effects (Tombaugh and McIntyre, 1992). Consistent with this, the meta-analysis of studies using depression as a binary predictor revealed that effect sizes are significantly smaller for studies using the MMSE than studies using neuropsychiatric batteries. It is therefore possible that the widespread use of the MMSE may have resulted in an underestimation of the association between affective disorders and decline in cognitive state in healthy older adults over time. One further limitation is that excluding cognitive impairment and dementia at baseline does not completely rule out the possibility of reverse causality.

Beyond this, included studies used different approaches and instruments to assess affective problems. Research suggests that there is low overlap among different scales of depression and anxiety, with content analysis suggesting that different types of assessments may capture different symptoms (Fried, 2017). It is therefore possible that studies included in this review are not entirely comparable on the basis that the methods of assessing depression are heterogeneous and may each be capturing different kinds of symptoms. Beyond this, there were also very few studies which met our inclusion criteria which examined the association between anxiety and decline in cognitive state, meaning that a quantitative meta-analysis was not possible. Moreover, an additional limitation is that as there were no studies investigating comorbidity between anxiety and depression. Finally, many of the studies did not report separate effect sizes for different types of symptoms of affective problems (e.g. negative affect symptoms, somatic symptoms, etc.), meaning we could not look at how different symptoms of affective problems may influence decline in cognitive state in the current study.

### Plausible mechanisms

Three major hypotheses have been proposed to explain this observed association. The first states that affective problems may act as an aetiological risk factor for the subsequent decline in cognitive state, perhaps by lowering the threshold for manifesting decline (Butters *et al.*, 2008; Bennett and Thomas, 2014). The second hypothesis proposes that affective problems may act as a prodromal feature of dementia. Specifically, affective problems may manifest as an early clinical presentation of this disorder. Affective problems and decline in cognitive state may therefore be different symptoms of the same underlying condition (Panza *et al.*, 2010; Bennett and Thomas, 2014). The third hypothesis posits that affective problems and decline in cognitive state are separate processes but may share common risk factors and underlying neurobiological substrates (Djernes, 2006; Enache *et al.*, 2011; Bennett and Thomas, 2014). These hypotheses are not necessarily mutually exclusive and it is likely that multiple pathways and mechanisms underlie this relationship.

There are several biological and behavioural pathways which may be involved in the association between affective problems and decline in cognitive state. These include vascular disease, increased cortisol production leading to atrophy of the hippocampus (Geerlings and Gerritsen, 2017), increased deposition of *β*-amyloid plaques (Byers and Yaffe, 2011), inflammatory changes (Byers and Yaffe, 2011) and a decline in the levels and activities of neurotrophic factors (Royall *et al.*, 2017). A multiple pathways model has also been proposed by Butters *et al.* (2008), which posits that depression-associated cerebrovascular disease and glucocorticoid neurotoxicity may operate to decrease levels of brain and cognitive reserve, as well as interact with pathology of Alzheimer's disease, giving rise to the clinical manifestation of Alzheimer's disease and accelerated cognitive decline. Additional potential lifestyle and behavioural pathways associated with affective problems include educational attainment, social support, early life adversity and health behaviours such as exercise regime, alcohol consumption, smoking status and medication status. It is more likely that a complex interaction of biological and sociobehavioural mechanisms are involved in linking affective problems with cognitive decline, rather than one single aetiological determinant (da Silva *et al.*, 2013).

### Implications and future directions

Future research should focus on investigating whether effective treatment and management of affective problems may reduce risk of decline in cognitive state. Additionally, future reviews could focus on how affective problems are associated with decline in specific cognitive domains, such as memory, executive function and information processing speed. This information can help to elucidate the pattern of decline characteristic of individuals with a history of affective problems. The present review could not address the issue of comorbidity between depression and anxiety and how comorbidity is associated with subsequent decline in the mental state. Indeed, comorbidity of depression and anxiety disorders is common. It is estimated that around 50–60% of individuals who have experienced depression also have a history of anxiety disorder (Kessler *et al.*, 1996; Fava *et al.*, 2000). Additionally, it is believed that comorbidity of anxiety and depression may be related to higher symptom severity and persistence, as well as poorer functional outcomes (Angst *et al.*, 1999; Roy-Byrne *et al.*, 2000). For this reason, it is important for future research to address how comorbid depression and anxiety is associated with future cognitive decline, and whether comorbidity of these conditions may result in poorer cognitive outcomes than depression or anxiety in isolation. One additional question which remains unresolved is whether affective problems act as a risk factor for the accelerated decline in cognitive state or whether they are an early biomarker representing prodromal dementia. While we excluded studies where cognitive impairment was present at baseline, it is also known that dementia has a preclinical period of many decades (Sperling *et al.*, 2011). It is therefore possible that participants in included studies may have already built up substantial dementia pathology at baseline, even if cognitive symptoms were not yet apparent. Associations could therefore be due to reverse causality from subtle cognitive changes short of dementia. Future research should focus on distinguishing more clearly between these possibilities.

As average life expectancy lengthens and rapid demographic ageing occurs in populations worldwide, there is a dramatic predicted increase in the number of older adults living in our society (Oeppen and Vaupel, 2002; Lutz *et al.*, 2008). By 2030, it is estimated that approximately one in five people in England will be over the age of 65 (House of Lords, 2013). Given the predicted increase in population size of adults over the age of 65, as well as the poor outcomes and economic costs associated with a decline in cognitive state and impairment, it is important to identify life course risk factors for poorer late-life cognitive outcomes, for potential early intervention. These findings may have value in identifying individuals who may be at a greater risk of deterioration in cognitive function over time. It is possible that effective management and treatment of depression may reduce risk and improve cognitive outcomes within these individuals. However, there has also been some evidence to suggest there may be persisting neurocognitive disturbances even after remission of depression (Frasch *et al.*, 2000; Weiland-Fiedler *et al.*, 2004; Paelecke-Habermann *et al.*, 2005). Additionally, cognition may be an important treatment target for depression (Kaser *et al.*, 2017). Due to the high prevalence of depression in the population, these results are of great public health importance.

In conclusion, demographic ageing is occurring rapidly worldwide and the number of people living with dementia is expected to grow substantially in prevalence over the next thirty years. As such, focussing research on potentially modifiable life-course risk factors, such as affective problems, is of increasing importance. This review highlights the importance of affective problems, particularly depression, in this context.

## Supplementary material

For supplementary material accompanying this paper visit https://doi.org/10.1017/S0033291718001137. click here to view supplementary material
