# Supplementary material for: Affective problems and decline in cognitive state in older adults: a systematic review and meta-analysis
Source: Psychol Med. 2018 May 24;49(3):353–65. doi: 10.1017/S0033291718001137 (PMC6331688; doi:10.1017/S0033291718001137)

Supplementary Figure 1: Begg’s funnel plot for publication bias of studies using depression as a predictor of cognitive decline


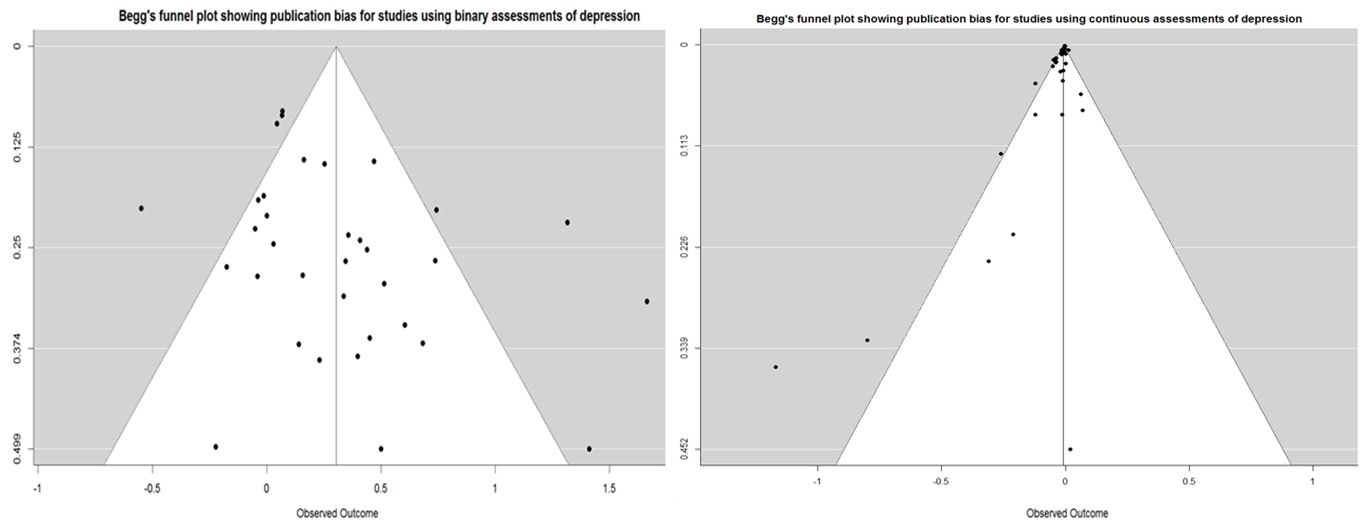

Supplement: Supplementary file 1 [file S0033291718001137sup001.docx]
